# Gene therapy: the end of the rainbow?

**DOI:** 10.1186/1758-3284-1-7

**Published:** 2009-03-30

**Authors:** Edward J Shillitoe

**Affiliations:** 1 Department of Microbiology & Immunology, SUNY Upstate Medical University, 750 East Adams Street, Syracuse, NY 13210, USA

## Abstract

The increased understanding of the molecular basis of oral cancer has led to expectations that correction of the genetic defects will lead to improved treatments. Nevertheless, the first clinical trials for gene therapy of oral cancer occurred 20 years ago, and routine treatment is still not available. The major difficulty is that genes are usually delivered by virus vectors whose effects are weak and temporary. Viruses that replicate would be better, and the field includes many approaches in that direction. If any of these are effective in patients, then gene therapy will become available in the next few years. Without significant advances, however, the treatment of oral cancer by gene therapy will remain as remote as the legendary pot of gold at the end of the rainbow.

## Introduction

Recent research into oral cancer has revealed a large amount of information about the nature of the disease. The details of many of the genetic changes are now available, raising the possibility that they could be reversed and that the growth of the tumors could be prevented. At the same time, information has accumulated about the oral viruses that could be used as delivery systems for the new treatments. Efforts to treat oral cancer in this way have thus been in progress for over 20 years. Despite this there is still no scientific evidence that oral cancer in humans can be managed by any form of genetic manipulation, or by the use of any viral vectors. Nevertheless, interest in the subject is maintained by promising advances in animals and by the unfortunate lack of progress in competing fields such as chemotherapy. This review surveys the most promising aspects of gene therapy that have emerged in the last two decades, and attempts to identify the areas in which the most effort should be invested.

### Approaches to gene therapy

#### Replacement of defective genes

In many cases of oral cancer some genes are defective, and replacing them with a normal variant is an obvious therapeutic strategy. The gene that has attracted most attention is p53, which is defective in about 50% of cases. Efforts to replace p53 have existed for 20 years, starting in cell culture and advancing through animal studies to phase I clinical trials [1]. The results have shown some promise in animals and the virus vector is well tolerated in humans. No results from adequately controlled trials are yet available. It is also unclear if the non-replicating virus vector is transducing a suitable proportion of tumor cells, or whether the normal p53 gene can indeed substitute functionally for a mutated gene in a tumor.

Another approach to therapy consists of suppressing genes that have become defective or are overexpressed. There are numerous examples of such genes, including regulators of apoptosis [2], genes from papillomaviruses [3], and the gene that encodes the receptor for Epidermal Growth Factor [4]. Methods of suppression have used antisense RNA, ribozymes, and siRNA, and delivery methods have included direct transfer, lipofection, and adenovirus vectors [5]. These studies have succeeded in reducing the growth and the malignant characteristics of tumor cells in vitro, but not in animals or patients.

#### Suicide gene therapy

Many anti-cancer drugs can be delivered as inactive precursors, and be activated by enzymes that are encoded by specific genes. If such genes can be provided to tumor cells only, then systemic delivery of the drug precursor could lead to an effective anti-tumor effect without side effects in other tissues. The most popular example of this approach has been to provide the gene for thymidine kinase from herpes simplex virus type 1 (HSV-1) to cells or tumors, followed by the pro-drug ganciclovir [6]. Another example is the gene CYP2B1 which activates cyclophosphamide to a toxic form [7]. This approach can kill oral cancer cells efficiently in culture and reduce the growth of tumors in animals [8] but no human clinical trial has demonstrated its efficacy.

### Viral Vectors

Although there are several non-viral methods of transferring genes to oral cancer cells, it is generally accepted that viruses provide the most efficient form of transfer. Early experiments used viruses that were mutated so as to prevent their replication, but more recent work has focused on viruses that can replicate in specific tumor cells.

#### Non replicating viral vectors

Early efforts at gene transfer were impeded by concerns that the virus vectors might cause infections or malignancies in the recipients. To prevent this, the vectors were prevented from replicating by removal of essential genes. The resulting viruses were still able to transfer marker genes to cells, but since they did not spread the markers were soon lost [9,10]. Clearly, if the marker was replaced by a therapeutic gene the therapeutic effect would be limited to the site of viral injection, which can only be a small proportion of a tumor.

#### Conditionally replication-competent vectors

A more efficient way to deliver toxic or therapeutic genes is in a virus that can replicate in a tumor cell but can not replicate in any other kind of cell. In the case of HSV-1 this can be achieved by deletion of viral genes that contribute to either neurotoxicity or to replication in normal cells, such as the genes gamma-1-34.5 and ribonucleotide reductase respectively. A viral mutant that lacks both of these genes and has been widely tested is named G207 [11]. Other mutants have been generated by changes in the genes UL56 or UL24 [12]. As a result of multiple deletions such as these, viral strains exist that can be injected directly to brain of herpes-susceptible primates without development of an infection [13]. Herpes viruses with these deletions can inhibit the growth of oral cancer cells that are growing as tumors in mice [14], but are not effective with every cell line [15].

Adenoviruses can also be manipulated to limit the tissues in which they replicate. An early example was the adenovirus Onyx015, which has a deletion of the E1B gene that increases its tumor specificity. This virus was tested in early clinical trials, and appeared to show a therapeutic effect in about 14% of patients [16]. Further clinical tests were discontinued, but the virus is available for treating oral cancer in China [17]. This probably represents the only example of gene therapy for any condition that currently exists outside of a clinical trial.

A different approach to change the tissue specificity of a virus is to replace the promoter of an essential gene with a promoter that is particularly active in specific tumors. Gene promoters that have been tested include those from liver [18], from soft tissues and bone [19] and those that respond to specific oncogenes [20,21]. For oral cancer the only tumor-related gene promoter that appears to have been used is one derived from a human papillomavirus. Papillomavirus promoters show a high level of specificity for oral cancer cells [22] and a strain of HSV-1 in which replication is driven by a papillomavirus promoter is under evaluation [23].

#### Safety of viral vectors

Although earlier concerns about the safety of viral vectors are now seen as having been exaggerated, there are still potential side effects. Widespread publicity has been given to a fatal reaction to a high dose of adenovirus in a trial of gene therapy for a liver enzyme deficiency [24], as well as to the unfortunate induction of leukemia in a small group of children who were being treated for a congenital immune deficiency [25]. Overall, however, given the large number of patients who have been entered into various gene therapy clinical trials, the use of viral vectors appears to be extremely safe.

#### Enhancement of viral virulence

The effectiveness of conditionally-replicating viruses can be increased still further by arming them with genes that encode toxic functions. Recent examples of such genes include those that encode cytokines such as GM-CSF [26] and those that specify formation of syncytia [27]. The addition of the gene for interleukin 12 has been found to increase the effect of treating oral cancer in mice with suicide gene therapy [8].

#### Immune suppression

All of the viruses that have been used for experimental gene therapy are as antigenic as the wild type viruses, and thus they are can stimulate immune responses that affect their function. To prevent this, various efforts have been made to suppress immunity and increase the effects of the virus.

Cyclophosphamide increases the anti-tumor effect of HSV-1 in rodent models of glioblastoma multiforme, and this has been attributed to reductions in complement and natural antibody which normally act together to reduce the replication of the virus [28,29]. In tumors, cyclophosphamide can reduce infiltration by phagocytic cells and this increases the proportion of tumor cells that are infected [30]. In some situations, in contrast, the immune response might be actually helpful in the elimination of infected tumors. Cyclophosphamide actually reduced the anti-tumor effect of the virus in a rodent model of malignant melanoma [31].

For oral cancers there are few data available on the role of the immune response in the effects of oncolytic viruses. We have tested the effects of HSV-1 on oral cancers in strains of mice that lacked several different components of innate and acquired immunity. The virus was no more effective in any strain [32], which implies only a minor role for anti-viral immunity in oral cancer.

#### Tissue permeabilization

The failure of viruses to spread through solid tumors might be due, in part, to the density of the tissue. In that case, any approach that loosens the tissue might allow more of the tumor to become infected. The injection of proteases such as collagenase or trypsin into experimental glioblastomas before injection of an adenovirus has led to better therapeutic result in one study [33]. The enzyme hyaluronidase also increases the anti-tumor effect of adenoviruses [34] and collagenase has been reported to allow oncolytic HSV-1 to infect more cells in a malignant melanoma [35]. Similarly, the enzyme relaxin can digest fibers in connective tissue and it is possible to clone the gene that encodes relaxin into adenoviruses [36]. Another approach to induce oncolytic HSV-1 to spread through a tumor more effectively is to induce apoptosis in the tumor cells [37] although premature apoptosis could also inhibit the replication of the virus.

#### Concurrent therapeutic drugs

It is a standard practice in cancer therapy to combine two or more agents, because this often produces an additive or synergistic anti-tumor effect. If the side-effects of the agents are different, then this approach can minimize the unwanted effects of treatment. A similar approach has been investigated in the use of oncolytic viruses (Table 1) [12,38-46]. In animal models, drugs that have increased the anti-oral cancer effects of replicating viruses include cisplatin [38,47], Hexamethylene bisacetamide [43] and cyclophosphamide [42]. In a clinical trial of the adenovirus Onyx015 for treatment of oral cancer, the virus was given in combination with cisplatin or 5-FU [48].

**Table 1:** Drugs that increase the effects of oncolytic viruses

Drug	Enhanced anti-tumor effect in vivo^1^	Reference^2^
Cisplatin	Yes – Oral cancer	[38]
Cyclophosphamide	Yes – Oral cancer	[42]
Estrogen	No	[45]
5-FU	No	[40]
Gemcitabine	No	[40]
Hexamethylene bisacetamide	Yes – Oral cancer	[43]
Mitomycin C	Yes – Gastric cancer	[46]
N-acetylcysteine	No	[44]
Trichostatin A	No	[41]
Vincristine	Yes – Rhabdomyosarcoma	[39]

^1 ^Although each of the drugs shown here has been found to increase the growth-inhibiting effects of adenovirus or HSV-1-vectors in cell culture, only some have been shown to increase the anti-tumor effect in animal models.

^2 ^Only one representative reference is shown for each drug, although other laboratories have reported similar findings in many cases.

Surprisingly, there is little or no acknowledgement among those who have used it that the combination of viruses and cytotoxic drugs should, in fact, be expected to fail. Viruses almost always replicate better in cells that are healthy and growing rapidly. Therefore any cytotoxic drug should reduce the effects of a virus rather than enhance them, and combinations should be less effective than either agent alone. One reason that such combinations can be effective might be that they have been demonstrated only in mutant viruses that lack some essential function. The drug might stimulate the tumor cells to express that function and complement the viruses deficiency. For example, some drugs induce DNA repair functions including the expression of the gene GADD34 that can substitute for the viral gene gamma-1-34.5 [49]. In other cases the mode of enhancement is not known. Also unclear is whether any drug can increase the effects of attenuated mutants to the extent that they become more potent than the equivalent wild-type virus. Even wild-type viruses have only limited effects on oral cancers [42] and an anti-tumor effect that is better than that of the wild-type virus is necessary if oncolytic viruses are to be truly successful.

### The future of gene therapy

Many approaches to gene therapy of oral cancer now exist in the laboratory, and some have been tested in human patients. However, there seems to be no evidence so far that any gene therapy approach can be expected to be as good or better than conventional approaches to treatment. This is largely due to the lack of any model in which the virus replicates and spreads until the entire tumor is infected and all cells are destroyed. The field, nonetheless, continues to advance and new approaches are continually being brought forward for evaluation. Thus we can expect much more data to emerge over the next few years.

Gene therapy is sometimes seen as the pot of gold at the end of the rainbow. However, it must be remembered that not only are rainbows intangible, but the very existence of leprachauns and the pot of gold that they hide at the end is seriously doubted. Whether gene therapy belongs in the same category is unknown and significant improvements are needed to prevent the topic from fading into the category of appealing legends.

## Competing interests

The author declares that they have no competing interests.
